# Toenail selenium concentrations and bladder cancer risk in women and men

**DOI:** 10.1038/sj.bjc.6602788

**Published:** 2005-09-20

**Authors:** D S Michaud, I De Vivo, J S Morris, E Giovannucci

**Affiliations:** 1 Department of Epidemiology, Harvard School of Public Health, Boston, MA, USA; 2 Channing Laboratory, Department of Medicine, Brigham and Women's Hospital and Harvard Medical School, Boston, MA, USA; 3 Research Reactor Facility, University of Missouri, Columbia, MI, USA; 4 Department of Nutrition, Harvard School of Public Health, Boston, MA, USA

**Keywords:** toenail selenium, bladder cancer, cohort study

## Abstract

Prediagnostic selenium concentrations measured in archived toenails were inversely associated with bladder cancer risk in women (*P* for trend=0.02), but not in men, in a nested case–control study of 338 cases and 341 matched controls. These findings may be due to chance and more studies are needed to determine whether associations between selenium and bladder cancer risk differ by sex.

Data from animal studies, observational studies and clinical trials suggest that selenium is anticarcinogenic and can reduce the risk of a number of different cancers (Combs, 2005). Five studies, each with fewer than 36 bladder cancer cases, reported inconsistent findings for selenium measured in serum (Nomura *et al*, 1987; Helzlsouer *et al*, 1989), toenails (Knekt *et al*, 1990; Garland *et al*, 1995), or given as supplements in an intervention trial (Clark *et al*, 1996). In two large cohort studies with selenium measurements from prediagnosic toenail samples, no association was observed with bladder cancer risk among male smokers (Michaud *et al*, 2002), but a strong inverse association was reported in a study conducted in the Netherlands (Zeegers *et al*, 2002).

Of the two largest studies to date, only 59 women were available for analysis in one study (Zeegers *et al*, 2002) and the other study consisted only of men (Michaud *et al*, 2002). We examined the sex-specific association between prediagnostic toenail selenium and bladder cancer risk using data from two large cohort studies.

## MATERIALS AND METHODS

The Health Professionals Follow-Up Study (HPFS) and the Nurses' Health Study (NHS) cohorts provided data for our analyses of toenail selenium concentrations and bladder cancer risk. Details on these two cohorts have been previously published (Michaud *et al*, 2001). Toenail clippings were obtained from 33 737 HPFS participants in 1987 and from 68 213 NHS participants in 1983. At baseline, participants of these cohorts provided information on disease history, history of cigarette smoking, medication use, weight and height. In addition, smoking status is updated biennially. The National Death Index was used to determine vital status for nonrespondents, and the remaining nonrespondents were assumed to be alive and at risk for bladder cancer incidence.

Participants report new medical conditions on a biennial basis. Self-reported bladder cancers are confirmed by review of medical records (87%) or with an additional letter or phone call. Similar procedures are followed when the National Death Index lists a previously unreported bladder cancer for a deceased participant.

Participants with bladder cancer were eligible for this analysis if the cancer diagnosis occurred after the toenail samples were obtained and they had no prior history of cancer (other than nonmelanoma skin cancer). From the time of the toenail collection through 2000, 338 incident cases with toenail samples were available for the selenium analyses (222 men; 116 women). Controls were matched (1 : 1) on gender, age (within 1 year), smoking status (current, past, never, unknown) and month of toenail collection. In addition, controls had to be cancer free (other than nonmelanoma skin cancer) at the time of the bladder cancer diagnosis in the matched case.

Selenium concentrations were measured in two batches by Dr Morris at the University of Missouri Research Reactor, Columbia, MO, USA, using neutron activation analysis (NAA) (Baskett *et al*, 1995). For quality control, selenium was measured in NIST SRM 1577, giving 1.088±0.065 *μ*g g^−1^ (*n*=24, in the first batch) and 1.108±0.031 *μ*g g^−1^ (*n*=8, in second batch) *vs* a certified value of 1.1±0.1. The CV% for these quality controls was 6.0 and 2.8 for the first and second batches, respectively. In addition, we obtained a CV% of 5.7 for 24 internal controls (split toenail samples), which were blinded to the laboratory.

Odds ratios (OR) and 95% confidence intervals (CI) were estimated using conditional logistic regression models. Quartiles of selenium concentrations were created according to the distribution of the controls for each sex, separately. A continuous variable for pack-years of smoking was additionally included in all models. As a secondary analysis, we performed analyses with a 4-year lag to exclude preclinical cases at baseline. All *P*-values are based on two-sided statistical tests. We performed tests for trend by assigning the median value to each category and modelling this variable as a continuous variable.

## RESULTS

Mean selenium toenail concentrations were higher in men than women (0.896 *vs* 0.787 *μ*g g^−1^, among controls, respectively), but similar among cases and controls for each sex (Table 1). Even though cases and controls were matched on smoking status (never, past, current), pack-years of smoking (among past and current smokers) were higher among cases than controls. Among women, menopausal status and age at menopause were similar in cases and controls.

In a conditional regression model, higher toenail selenium concentrations were associated with significantly lower risk of bladder cancer in women (*P* for trend=0.02), but not in men (Table 2).

Median selenium concentrations varied by smoking status (HPFS (*μ*g g^−1^): never 0.825, past 0.814, current 0.739; NHS (*μ*g g^−1^): never 0.777, past 0.785, current 0.716). Among women, the strongest association between selenium and bladder cancer was observed among past smokers (RR=0.17, 95% CI=0.04–0.74 for the highest tertile of selenium, compared to the lowest); among men, smoking status did not significantly modify the associations (data not shown).

Removing cases diagnosed with bladder cancer within the first 4 years of follow-up strengthened the inverse association in women (RR=0.27, 95% CI=0.10–0.74, highest *vs* lowest quartile), but resulted in a positive association in men (RR=1.53, 95% CI=0.71–3.28, highest *vs* lowest quartile).

## DISCUSSION

In this nested case–control, a strong inverse association was observed between toenail selenium concentrations and bladder cancer risk in women, but no association was observed in men. The association, in women, was strongest among past smokers.

In the largest study to date on toenail selenium concentrations and bladder cancer, only 59 cases (out of 431 cases) were women and associations were not presented separately by sex (Zeegers *et al*, 2002). In that study, conducted in the Netherlands between 1986 and 1992, mean toenail selenium concentrations were substantially lower than ours, in both men and women (0.536 for men, 0.568 for women). A relative risk of 0.67 (95% CI=0.46–0.97) was reported for the highest *vs* lowest quintile of selenium toenail level; the strongest association was among those who recently quit smoking (⩽10 years) and no associations were noted for never and current smokers. Our findings for women were similar to these as the inverse associations were also strongest among past smokers.

Data from animal studies, epidemiologic data and human prevention trials suggest that selenium is an important chemopreventive agent (Combs, 2005). Although the exact mechanism by which selenium prevents cancer is unknown, multiple mechanisms are likely to be involved. At least part of the antimutagenic effect of selenium is mediated by Se-dependent enzymes such as glutathione peroxidases and thioredoxin reductases, which protect DNA from oxygen radicals (Combs and Gray, 1998). In Se-adequate individuals, the anticarcinogenic effect of selenium may be mediated by selenium metabolites which can influence carcinogen metabolism, immune function, tumour growth, apoptosis induction (Combs and Gray, 1998) and DNA methylation (Schrauzer, 2000).

The different results observed in men and women may be due to a chance inverse association in women. Alternatively, a possible explanation for the lack of association in men in our study could be that selenium offers no additional protection within the observed range of selenium concentrations. Mean selenium concentrations were slightly higher in men than women in our study and substantially higher than those previously reported in the bladder study conducted in the Netherlands (Zeegers *et al*, 2002). The large sex differences in incidence rates that exist for this cancer suggest that important etiologic differences exist between men and women; thus, it is plausible that the association between selenium and bladder cancer varies by sex. An alternative possibility is that the difference is due to the timing of the toenail collection. On average, women in the NHS provided toenails when they were 8½ years younger than when men in the HPFS provided toenails (Table 1), and consequently this difference may have provided a better measure of early exposure for women.

A limitation to our study includes possible measurement error when assessing toenail selenium concentrations, which may have resulted in the null finding in men. However, because toenails from the two different cohorts were analysed at the same time for each of the two batches, and CV% were good for both batches, it is unlikely that this could explain the different results by sex.

Strengths of this study include the largest number of female bladder cancer cases to date with selenium data, pre-diagnostic measurements, use of toenails (where selenium is more time-integrated), detailed data on potential confounders, 1 : 1 matching by known confounders and high follow-up rates in the two cohorts used to select cases and controls.

In summary, we observed a statistically significant inverse association between prediagnostic selenium toenail concentrations and risk of bladder cancer in women, but not in men. To date, this is the largest study to report findings on selenium concentrations and bladder cancer risk in women. Future studies are needed to confirm our findings.

## Figures and Tables

**Table 1:** Prediagnostic toenail selenium level and baseline characteristics of nested bladder cancer cases and matched controls from the NHS and HPFS cohort studies

	**Mean (s.d.) or %**	
	**Cases**	**Controls**
*Men*		
No. of subjects	222	224
Toenail selenium level (*μ*g g^−1^)^a^	0.897 (0.53)	0.896 (0.58)
Median	0.814	0.805
Age (years)^b^	62.3 (8.6)	62.3 (8.6)
Smoking (%)^b^		
Never	26.1	25.9
Past	53.6	54.0
Current	16.7	16.5
Missing	3.6	3.6
Pack-years^c^	34.1 (22.9)	29.6 (23.5)
		
*Women*		
No. of subjects	116	117
Toenail selenium level (*μ*g g^−1^)	0.802 (0.50)	0.787 (0.19)
Median	0.733	0.763
Age (years)^b^	53.7 (6.0)	53.7 (6.1)
Smoking (%)^b^		
Never	26.5	26.5
Past	23.9	24.8
Current	48.7	47.9
Missing	0.9	0.9
Pack-years^c^	31.7 (22.9)	24.6 (17.8)
Premenopausal	21	22
Postmenopausal	74	71
Dubious/missing	5	7
Age at menopause^d^	48.9 (4.2)	49.4 (3.5)

NHS=Nurses' Health Study; HPFS=Health Professionals Follow-Up Study; s.d.=standard deviation.

a Based on 221 cases and 223 controls.

b Matched factors (age at time of toenail collection, smoking status in 1986 for men and 1980 for women).

c Among past and current smokers.

d Among natural postmenopausal women (55 cases and 49 controls).

**Table 2:** Relative risks (95% confidence intervals) of bladder cancer associated with prediagnostic toenail selenium concentrations in a nested case–control study of the NHS and HPFS cohorts

	**1**	**2**	**3**	**4**	***P*-value, trend test**
*Men*					
Median selenium level (*μ*g g^−1^)^a^	0.660	0.765	0.863	0.990	
Case/control	52/55	54/57	53/55	62/56	
RR^b^	1.0	1.04	1.05	1.19	0.50
RR^c^	1.0	1.09	1.08	1.17	0.61
95% CI	Ref.	0.59–2.01	0.59–2.00	0.66–2.07	
					
*Women*					
Median selenium level (*μ*g g^−1^)^a^	0.626	0.716	0.800	0.933	
Case/control	37/28	35/31	23/29	21/29	
RR^b^	1.0	0.80	0.49	0.42	0.04
RR^c^	1.0	0.73	0.49	0.36	0.02
95% CI	Ref.	0.35–1.55	0.21–1.16	0.14–0.91	

NHS=Nurses' Health Study; HPFS=Health Professionals Follow-Up Study; RR=relative risk; CI=confidence interval.

a Quartile cutpoints for men: <0.723; 0.723–0.805; 0.806–0.911; >0.911; and women: <0.687; 0.687–0.763; 0.764–0.839; >0.839.

b Conditional regression analysis (matched factors: age, month of toenail collection, smoking status).

c Additionally controlling for pack-years smoked (continuous) and current heavy cigarette smoking at baseline (25+ per day).
